# Analgesic effect of external oblique intercostal block in laparoscopic cholecystectomy: A retrospective study

**DOI:** 10.1515/med-2024-1068

**Published:** 2024-12-06

**Authors:** Shuai Yi, Dan Li, Xin-lei Zhang, Fen-yu Duan, Han Gao, Ming-jian Kong

**Affiliations:** Department of Anesthesiology, The Second Affiliated Hospital of Xuzhou Medical University, Xuzhou, 221006, Jiangsu, China; Department of Anesthesiology, The Second Affiliated Hospital of Xuzhou Medical University, No. 32 Meijian Road, Xuzhou, 221006, Jiangsu, China

**Keywords:** external oblique intercostal block, laparoscopic cholecystectomy, postoperative analgesia, postoperative recovery

## Abstract

**Objective:**

The aim of this study was to assess the impact of the external oblique intercostal block (EOIB) on early postoperative pain in patients who underwent laparoscopic cholecystectomy.

**Methods:**

120 patients were divided into two groups: the EOIB group (Group E) and the control group (Group C). The assessed variables were mainly intraoperative remifentanil usage, numerical rating scale (NRS) pain scores at 0, 1, 2, 4, 6, 12, and 24 h postoperatively, cumulative fentanyl consumption within 24 h postoperatively and within the first-hour post-anesthesia care unit.

**Results:**

Remifentanil consumption during surgery was significantly reduced in Group E compared to Group C. Postoperative fentanyl requirements were also lower in Group E at 1 and 24 h after surgery. Furthermore, Group E demonstrated significantly lower NRS scores at 0, 1, 2, 4, and 6 h postoperatively and a reduced need for rescue analgesia compared to Group C. However, at 12 h post-surgery, Group E’s NRS scores were slightly higher than Group C’s.

**Conclusion:**

The EOIB is associated with reduced pain within the first 24 postoperative hours following laparoscopic cholecystectomy.

## 1 Introduction

Laparoscopic cholecystectomy is the standard surgical approach for gallbladder removal, involving the creation of port sites through incisions below the xiphoid process, above the umbilicus, and below the right costal margin. While this minimally invasive technique reduces postoperative pain, it can still result in moderate-to-severe pain [1]. Approximately 21–41% of patients undergoing laparoscopic cholecystectomy experience pain that delays early discharge [2], highlighting the necessity for adequate analgesia after minimally invasive surgery.

Opioids are the most extensively utilized analgesics in clinical practice. However, their overuse can lead to adverse effects such as respiratory depression, sedation, constipation, nausea, and vomiting, with a higher potential for addiction compared to other medications [3]. Peripheral nerve blocks are widely applied for analgesia in lower limb, thoracic, and abdominal surgeries due to their minimal impact on circulation, respiration, coagulation, and consciousness, coupled with their ease of administration and significant analgesic effects [3,4,5]. These nerve blocks constitute an essential component of multimodal systemic analgesia. Fascial plane blocks involve the injection of local anesthetics into the fascial planes between target muscles, where they diffuse and block sensory afferent nerves, achieving analgesia [6].

The transverse abdominal plane block (TAPB) is extensively utilized in abdominal surgery. Studies have indicated that, in the context of postoperative analgesia following laparoscopic cholecystectomy, there is no significant difference in postoperative morphine consumption or numerical rating scale (NRS) scores between continuous TAPB, single-shot TAPB combined with intravenous patient-controlled analgesia (PCIA), and intravenous PCIA alone [7]. This suggests that the analgesic efficacy of the lateral approach TAPB in laparoscopic cholecystectomy is comparable to that of intravenous analgesia alone.

In 2019, Hamilton et al. introduced the external oblique intercostal block (EOIB), with initial cadaveric studies demonstrating staining of the lateral cutaneous branches of the intercostal nerves from T6 to T10 [8,9]. In 2021, Elsharkawy et al. performed bilateral EOIBs using dye and 0.25% bupivacaine in both cadavers and patients. The staining of the lateral and anterior branches from T7 to T10 was consistent in cadavers, while in patients, a sustained block from T6 to T10 was observed along the anterior axillary line, and from T6 to T9 along the mid-clavicular line [10]. Liotiri et al. conducted single-shot EOIBs in two patients undergoing laparoscopic hepatectomy and found it provided effective postoperative analgesia [11]. O’Donovan et al. implemented continuous EOIB via a catheter in a patient undergoing open cholecystectomy, resulting in a resting visual analog scale pain score of 1 and a score of 3 with movement [12]. Thus, the EOIB may represent a promising fascial plane block for laparoscopic cholecystectomy.

The aim of this study is to evaluate whether EOIB can provide effective analgesia after laparoscopic cholecystectomy, enhance patient recovery rate and quality, and provide clinical evidence for the application of EOIB.

## 2 Materials and methods

### 2.1 Study design and patient population

This retrospective study analyzed data from patients who underwent laparoscopic cholecystectomy at the Second Affiliated Hospital of Xuzhou Medical University from January 2023 to November 2023. The sample size was not predetermined, and the study sample comprised patient data collected during the study period. Inclusion criteria were as follows: (1) elective laparoscopic cholecystectomy with a drain placed in the right lateral abdominal wall between January 2023 and November 2023; (2) American Society of Anesthesiologists (ASA) physical status classification I–II; (3) age between 18 and 65 years; and (4) absence of significant psychiatric disorders. Patient data were retrieved from medical records, including observation records provided by nurses in the post-anesthesia care unit (PACU) and general surgical ward. Exclusion criteria included: (1) patients with severe chronic cardiovascular, pulmonary, or neurological diseases; (2) history of chronic pain; (3) long-term or recent use of opioids; (4) history of allergy to anesthetic drugs; (5) patients discharged before surgery completion or those requiring conversion to open surgery; and (6) history of previous abdominal or laparoscopic surgery. Patients were divided into two groups: the EOIB group (Group E), which received EOIB after anesthesia induction; and the control group (Group C), which received intravenous analgesics 30 min preoperatively.

### 2.2 Anesthesia management

Standard general anesthesia was administered for routine laparoscopic cholecystectomy. Propofol at 2.5 mg/kg, fentanyl at 2 μg/kg, and rocuronium at 0.6 mg/kg were used for intravenous induction. Cuffed endotracheal tubes of sizes 6.5–7.0 for females and 7.0–7.5 for males were utilized. Anesthesia was maintained with 1.0–1.5% sevoflurane and a remifentanil infusion at 0–0.3 μg/(kg min) until the conclusion of skin suturing. Patients were ventilated through an endotracheal tube with a mixture of oxygen and air in a 1:1 ratio, with end-tidal carbon dioxide levels maintained between 35 and 45 mmHg. Remifentanil dosage was adjusted to keep the bispectral index within 40–60, systolic blood pressure fluctuations within 20% of baseline, and heart rate between 55 and 75 beats per minute (bpm).

**Figure 1:**
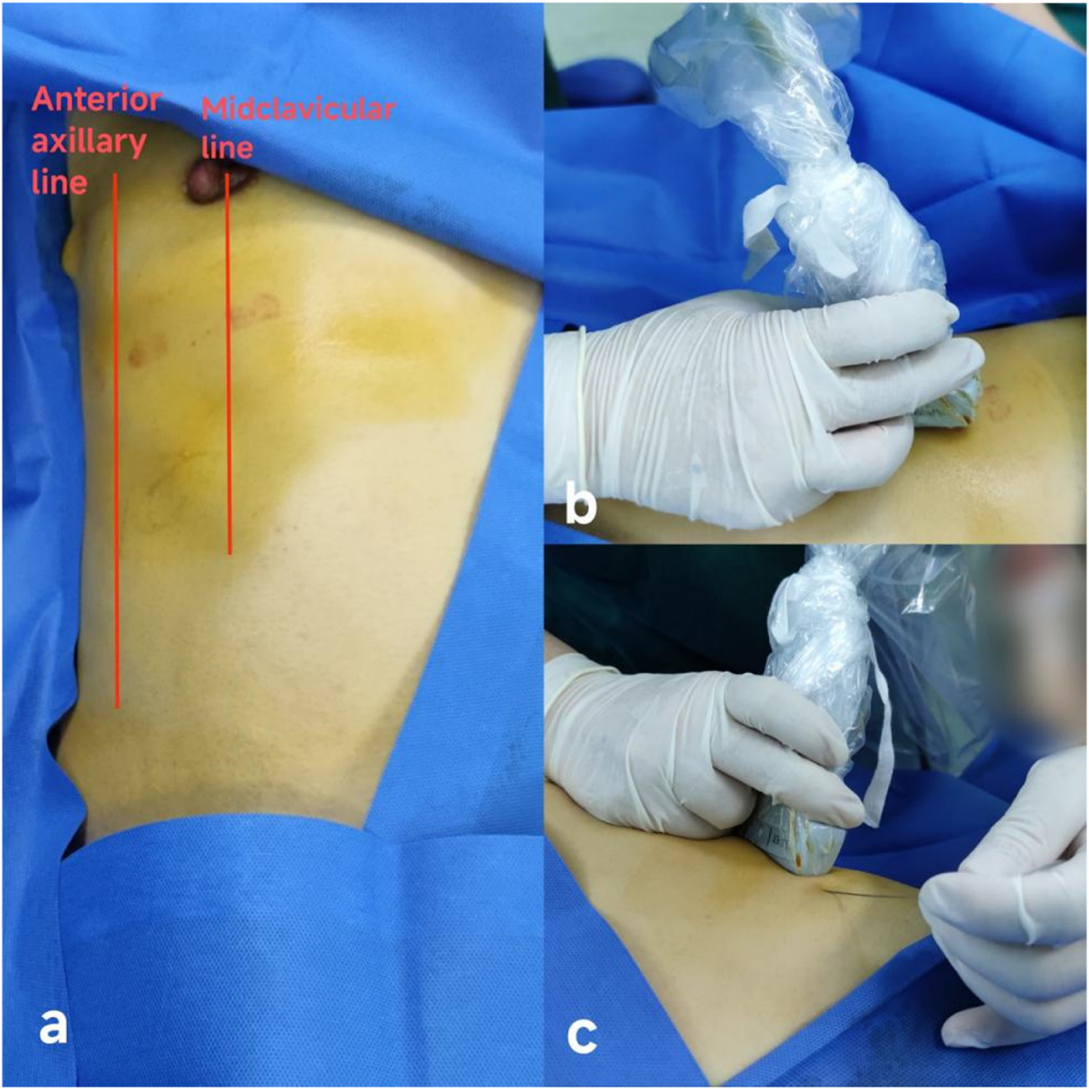
(a) Location of midclavicular line and anterior axillary line; (b) placement of ultrasound probe; and (c) position and direction of needle insertion. The implementation process of EOIB.

**Figure 2:**
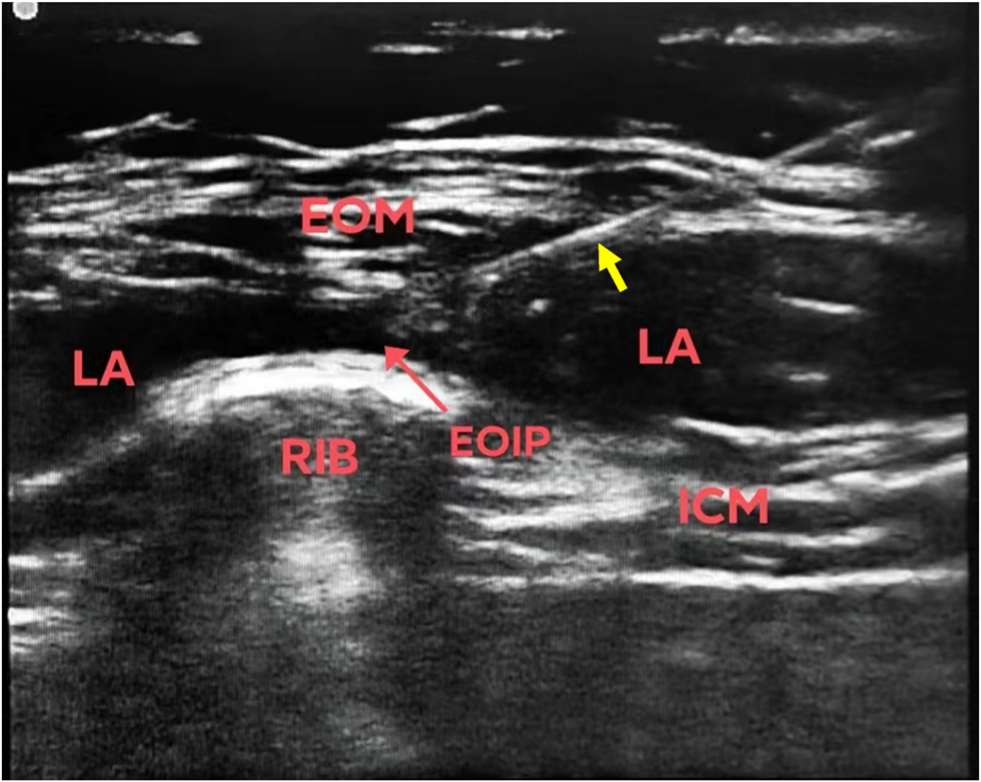
The location of the puncture needle and the spread of the local anesthetic (yellow arrow points to the body of the needle). LA: local anesthetic; ICM: intercostal muscle; EOM: external oblique muscle; EOIP: external oblique intercostal plane.

For patients receiving the EOIB (Group E), all blocks were performed unilaterally on the right side by the same anesthesiologist trained in ultrasound-guided nerve blocks. After donning gloves, disinfection, and draping with a sterile sheet, a linear array transducer (12–15 MHz, Sonosite) with sterile ultrasound gel was positioned in the sagittal plane between the midclavicular line and the anterior axillary line (Figure 1a). The seventh rib was first identified, and then the transducer was rotated medially with the proximal end slightly angled medially (Figure 1b) to obtain a mid-axillary sagittal oblique view and short-axis view of the rib. Approximately 1–2 cm medial to the anterior axillary line, the following layers were sequentially identified from superficial to deep: subcutaneous tissue, external oblique muscle, intercostal muscles, pleura, and lung. The needle entry point was located on the medial side of the anterior axillary line at the level of the seventh rib, with the needle tip directed towards and on the surface of the rib. A hydrodissection with 2–3 ml of normal saline was performed first, followed by the injection of 30 ml of 0.375% ropivacaine into the plane (Figure 2); the spread of local anesthetic along the plane was monitored with ultrasound. For the control group patients (Group C) receiving intravenous analgesics, ketorolac tromethamine 30 mg and tramadol 1 mg/kg were administered intravenously 30 min before skin closure.

### 2.3 Study outcomes

The primary outcomes were the pain scores and fentanyl consumption within the first hour after patient transfer to the PACU. The time of patient transfer to the PACU was designated as 0 h. The pain was assessed using the NRS, where 0 indicates no pain and 10 indicates the worst possible pain. NRS scores were collected at 0, 1, 2, 4, 6, 12, and 24 h postoperatively. Pain management involved PCIA with fentanyl administered at a dose of 15 μg per demand, with a lockout time of 10 min and no basal infusion. Rescue analgesia was provided in the form of intravenous parecoxib 40 mg. Data collected from the medical records of patients in both groups included intraoperative remifentanil consumption, cumulative fentanyl consumption within the first 24 h postoperatively, fentanyl consumption within the first hour in the PACU, the need for rescue analgesia, the incidence of postoperative nausea and vomiting in both groups, and the time to first ambulation and first food intake in the ward.

### 2.4 Statistical analysis

Statistical analysis was performed using SPSS 26.0 (SPSS Inc., Chicago, IL, USA). The Shapiro–Wilk test was used to determine the normal distribution for continuous data, with data expressed as mean ± standard deviation ( \[\bar{X}\pm \hspace{0.1em}\text{SD}]\] ) for continuous variables and percentage (%) for categorical variables. For normally distributed continuous variables, the Student’s *t*-test test was used for comparison between groups. For non-normally distributed continuous data, comparisons between groups were performed using the Mann–Whitney *U* test. Categorical variables were compared between groups using the chi-square test or Fisher’s exact test. All tests were two-sided, and *P* < 0.05 was considered statistically significant.


**Ethical approval:** This study was approved by the Institutional Review Board of the Second Affiliated Hospital of Xuzhou Medical University (Approval No.: [2023]120104). As a retrospective analysis, the requirement for individual informed consent was waived by the ethics committee.

## 3 Results

We reviewed the records of 296 patients who underwent laparoscopic cholecystectomy between January 2023 and November 2023. A total of 176 patients were excluded from the study; 92 did not meet the inclusion criteria, and 84 had incomplete postoperative pain management records. Ultimately, 120 patients with complete record data were included in the study.

Patients were divided into two groups: those who received the EOIB after anesthesia induction (Group E; *n* = 53) and those who did not receive any nerve block and only received intravenous analgesia 30 min before the end of surgery (Group C; *n* = 67). The baseline demographic data for both groups are presented in Table 1. There were no statistically significant differences between the groups in terms of age, gender distribution, ASA classification, and surgical duration.

**Table 1:** Baseline demographic data for patients (*n* = 120)

Characteristic	Group C	Group E	Statistical analysis
	*n* = 67	*n* = 53	
Age, years	46.85 ± 3.07	47.13 ± 3.27	*p* = 0.629
Sex			
Female	42 (62.7)	34 (64.2)	*p* = 0.869
Male	25 (37.3)	19 (35.8)	
ASA			
I	54 (80.6)	43 (81.1)	*p* = 0.941
II	13 (19.7)	10 (18.9)	
Duration of surgery, min	50.48 ± 4.11	51.38 ± 4.21	*p* = 0.20

Data presented as mean ± SD or *n* of patients (%).

No significant between-group differences (*P* > 0.05); normally distributed continuous variables were compared using Student’s *t*-test; continuous variables that were not normally distributed were compared using Mann–Whitney *U*-test; categorical variables were compared using *X* ^2^-test.

ASA, American Society of Anesthesiologists; PACU: post-anesthesia care unit.

Group E patients had significantly lower intraoperative remifentanil consumption (225.64 ± 21.19 μg vs 174.49 ± 11.69 μg, *P* < 0.01), fentanyl consumption at 1 h postoperatively (31.13 ± 14.67 μg vs 65.37 ± 13.23 μg, *P* < 0.01), and cumulative fentanyl consumption within the first 24 h postoperatively (267.45 ± 35.27 μg vs 323.28 ± 34.07 μg, *P* < 0.01) compared to Group C (Table 2). Patients in Group E had a shorter stay in the PACU than those in Group C (*P* < 0.01). NRS scores at 0, 1, 2, 4, and 6 h postoperatively were significantly lower in Group E than in Group C, while the NRS score at 12 h postoperatively was higher in Group E (*P* < 0.01) (Table 2), with no difference in NRS scores at 24 h (1.87 ± 0.70 vs 1.88 ± 0.71, *P* > 0.05) (Table 2). The proportion of patients in Group E requiring rescue analgesia was significantly lower than in Group C (*P* < 0.05) (Table 2), and the incidence of postoperative nausea and vomiting, as well as the time to first ambulation and first oral intake, was significantly lower in Group E than in Group C (*P* < 0.01) (Table 2).

**Table 2:** Clinical characteristics and status of recovery for patients (*n* = 120)

	Group C	Group E	Statistical
Characteristic	*n* = 67	*n* = 53	analysis^a^
NRS scores			
0 h (PACU)	3.88 ± 0.69	2.08 ± 0.83	*p* < 0.01
1 h(PACU)	3.15 ± 0.70	1.57 ± 0.57	*p* < 0.01
2 h	3.99 ± 0.70	0.92 ± 0.65	*p* < 0.01
4 h	3.24 ± 0.87	1.19 ± 0.76	*p* < 0.01
6 h	2.81 ± 0.66	2.38 ± 0.77	*p* = 0.004
12 h	2.07 ± 0.66	2.40 ± 0.49	*p* = 0.007
24 h	1.88 ± 0.71	1.87 ± 0.70	NS
Analgesic requirement			
No	38 (56.7)	41 (77.4)	*p* = 0.018
Yes	29 (43.3)	12 (22.6)	
PACU time, min	84.07 ± 4.40	70.58 ± 4.75	*p* < 0.01
Remifentanil consumption, μg	225.64 ± 21.19	174.49 ± 11.69	*p* < 0.01
Fentanyl consumption (1 h after arrival in PACU), μg	65.37 ± 13.23	31.13 ± 14.67	*p* < 0.01
Fentanyl consumption (24 h after operation), μg	323.28 ± 34.07	267.45 ± 35.27	*p* < 0.01
PONV			
No	47 (70.1)	46 (86.8)	*p* < 0.05
Yes	20 (29.9)	7 (13.2)	
Time to first ambulation, h	11.58 ± 2.12	8.89 ± 1.48	*p* < 0.01
Time to first food intake, h	15.75 ± 2.39	13.06 ± 2.00	*p* < 0.01

Data presented as mean ± SD or *n* of patients (%).

^a^Normally distributed continuous variables were compared using Student’s *t*-test; continuous variables that were not normally distributed were compared using Mann–Whitney *U*-test; categorical variables were compared using *X* ^2^-test.

NRS, numerical rating scale; PONV, postoperative nausea and vomiting; NS, no significant between-group differences (*P* > 0.05).

## 4 Discussion

Studies have demonstrated that postoperative pain following upper abdominal surgeries is more intense compared to that of lower abdominal procedures, with up to 72.8% of patients requiring rescue analgesia [13]. During laparoscopic cholecystectomy, trocars are inserted and ports are established below the xiphoid process, above the umbilicus, and along the subcostal margin on the right side. The pain from these incisions is primarily governed by the anterior and lateral cutaneous branches of the intercostal nerves T7–T11. Consequently, blocking the anterior and lateral cutaneous branches of these nerves is a crucial target for postoperative analgesia following laparoscopic cholecystectomy.

The TAPB is widely used in abdominal surgery, but literature suggests that it only blocks the T10–L1 nerve roots [14]. Ortiz demonstrated that bilateral TAPB provides no superior postoperative analgesic effect compared to local infiltration anesthesia in laparoscopic cholecystectomy [15], indicating that TAPB may be suitable only for lower abdominal surgeries. Hebbar introduced the oblique subcostal transversus abdominis plane (OSTAP) [16], and studies suggest that OSTAP provides sensory blockade that can reach up to the T8 [17], effectively alleviating pain from midline upper abdominal incisions. However, its efficacy in alleviating pain from lateral abdominal wall incisions is reportedly limited [18].

In 2019, Hamilton introduced the EOIB [9], and anatomical studies have shown that 20 ml of dye during EOIB can stain the lateral and anterior cutaneous branches of T7–T10. In this study, Group E showed a reduction of approximately 50% in fentanyl consumption within the first hour postoperatively, a decrease in the rate of rescue analgesia required, and a mean difference in NRS scores greater than 2 points at 2 and 4 h postoperatively. These findings suggest that EOIB can block both the anterior and lateral cutaneous branches of T7–T10, providing sensory blockade for both the anterior abdominal wall and the lateral abdominal wall, compensating for the shortcomings of TAPB and OSTAP. They also highlight the importance and necessity of peripheral nerve blocks as a component of multimodal analgesia. These results are consistent with studies by White and Coşarcan et al. [19,20]. The lower NRS scores in Group C at 12 h and the smaller difference in cumulative fentanyl consumption between groups at 24 h may be due to the rescue analgesic medications administered to Group C, which alleviated pain and reduced fentanyl consumption in this group. The similar NRS scores at 24 h in both groups may relate to the gradual resolution of the single-shot nerve block effect.

Nerve blocks reduce the activation of the neuroendocrine system by blocking nociceptive afferents, mitigate systemic inflammatory responses, maintain immune function, reduce organ damage, and decrease the incidence of postoperative complications [21]. Patients in Group E were able to eat and ambulate earlier, suggesting that EOIB can block afferent nerves, accelerate patient recovery, and reduce adverse reactions. In contrast, Group C consumed more fentanyl, resulting in a higher incidence of nausea and vomiting.

This study demonstrates that EOIB has a definite blocking range and analgesic effect, can reduce the perioperative consumption of opioids in patients undergoing laparoscopic cholecystectomy, lower postoperative NRS scores, and facilitate postoperative recovery, making it a superior analgesic method. Limitations of this study include its retrospective nature, with all data obtained from the PACU, ward, and postoperative pain management records, which may be inaccurate and subject to bias; the patients included were from a single center, which may introduce regional variations; the concentration and dosage of local anesthetic used were based on cadaveric studies for this block, and the optimal concentration and dosage of local anesthetic were not further investigated; this study did not differentiate between somatic and visceral pain in patients, with pain intensity assessed using the NRS. Further confirmation of the study’s conclusions and issues related to local anesthetics requires large-sample, prospective, double-blind studies.

## References

[1] Wills VL, Hunt DR. Pain after laparoscopic cholecystectomy. Br J Surg. 2000;87(3):273–84. 10.1046/j.1365-2168.2000.01374.x 10718794

[2] Rahimzadeh P, Faiz SHR, Salehi S, Imani F, Mueller AL, Sabouri AS. Unilateral right-sided ultrasound-guided erector spinae plane block for post-laparoscopic cholecystectomy analgesia: a randomized control trial. Anesth Pain Med. 2022;12(6):e132152. 10.5812/aapm-132152 PMC10016115 36938107

[3] Wick EC, Grant MC, Wu CL. Postoperative multimodal analgesia pain management with nonopioid analgesics and techniques: a review. JAMA Surg. 2017;152(7):691–7. 10.1001/jamasurg.2017.0898 28564673

[4] Power I, McCormack JG, Myles PS. Regional anaesthesia and pain management. Anaesthesia. 2010;65(Suppl 1):38–47. 10.1111/j.1365-2044.2009.06202.x 20377545

[5] Beverly A, Kaye AD, Ljungqvist O, Urman RD. Essential elements of multimodal analgesia in enhanced recovery after surgery (ERAS) guidelines. Anesthesiol Clin. 2017;35(2):e115–43. 10.1016/j.anclin.2017.01.018 28526156

[6] Chin KJ, Lirk P, Hollmann MW, Schwarz SKW. Mechanisms of action of fascial plane blocks: a narrative review. Reg Anesth Pain Med. 2021;46(7):618–28. 10.1136/rapm-2020-102305 34145073

[7] Choi Y-M, Byeon G-J, Park S-J, Ok Y-M, Shin S-W, Yang K. Postoperative analgesic efficacy of single-shot and continuous transversus abdominis plane block after laparoscopic cholecystectomy: A randomized controlled clinical trial. J Clin Anesth. 2017;39:146–51. 10.1016/j.jclinane.2017.03.050 28494892

[8] Hamilton DL, Manickam BP. Is a thoracic fascial plane block the answer to upper abdominal wall analgesia? Reg Anesth Pain Med. 2018;43(8):891–2. 10.1097/AAP.0000000000000838 30339615

[9] Hamilton DL, Manickam BP, Wilson MAJ, Abdel Meguid E. External oblique fascial plane block. Reg Anesth Pain Med. 2019;44(4):528–9. 10.1136/rapm-2018-100256 30635518

[10] Elsharkawy H, Kolli S, Soliman LM, Seif J, Drake RL, Mariano ER, et al. The external oblique intercostal block: anatomic evaluation and case series. Pain Med. 2021;22(11):2436–42. 10.1093/pm/pnab296 34626112

[11] Liotiri D, Diamantis A, Papapetrou E, Grapsidi V, Sioka E, Stamatiou G, et al. External oblique intercostal (EOI) block for enhanced recovery after liver surgery: a case series. Anaesth Rep. 2023;11(1):e12225. 10.1002/anr3.12225 PMC10139870 37124666

[12] O’Donovan B, Martin B. The novel use of an external oblique nerve catheter after open cholecystectomy. Cureus. 2021;13(2):e13580. 10.7759/cureus.13580 PMC8005590 33796423

[13] Li TT, Chang QY, Xiong LL, Chen YJ, Li QJ, Liu F, et al. Patients with gastroenteric tumor after upper abdominal surgery were more likely to require rescue analgesia than lower abdominal surgery. BMC Anesthesiol. 2022;22(1):156. 10.1186/s12871-022-01682-w PMC9125846 35606700

[14] Tran TMN, Ivanusic JJ, Hebbard P, Barrington MJ. Determination of spread of injectate after ultrasound-guided transversus abdominis plane block: a cadaveric study. Br J Anaesth. 2009;102(1):123–7. 10.1093/bja/aen344 19059922

[15] Ortiz J, Suliburk JW, Wu K, Bailard NS, Mason C, Minard CG, et al. Bilateral transversus abdominis plane block does not decrease postoperative pain after laparoscopic cholecystectomy when compared with local anesthetic infiltration of trocar insertion sites. Reg Anesth Pain Med. 2012;37(2):188–92. 10.1097/AAP.0b013e318244851b 22330261

[16] Hebbard PD, Barrington MJ, Vasey C. Ultrasound-guided continuous oblique subcostal transversus abdominis plane blockade: description of anatomy and clinical technique. Reg Anesth Pain Med. 2010;35(5):436–41. 10.1097/aap.0b013e3181e66702 20830871

[17] Lee TH, Barrington MJ, Tran TM, Wong D, Hebbard PD. Comparison of extent of sensory block following posterior and subcostal approaches to ultrasound-guided transversus abdominis plane block. Anaesth Intensive Care. 2010;38(3):452–60. 10.1177/0310057X1003800307 20514952

[18] Chen Y, Shi K, Xia Y, Zhang X, Papadimos TJ, Xu X, et al. Sensory assessment and regression rate of bilateral oblique subcostal transversus abdominis plane block in volunteers. Reg Anesth Pain Med. 2018;43(2):174–9. 10.1097/AAP.0000000000000715 29278604

[19] White L, Ji A. External oblique intercostal plane block for upper abdominal surgery: use in obese patients. Br J Anaesth. 2022;128(5):e295–7. 10.1016/j.bja.2022.02.011 35249704

[20] Cosarcan SK, Yavuz Y, Dogan AT, Ercelen O. Can postoperative pain be prevented in bariatric surgery? Efficacy and usability of fascial plane blocks: a retrospective clinical study. Obes Surg. 2022;32(9):2921–9. 10.1007/s11695-022-06184-9 35776242

[21] Novak-Jankovič V. Regional anaesthesia in thoracic and abdominal surgery. Acta Clin Croat. 2019;58(S1):96–100. 10.20471/acc.2019.58.s1.14 PMC6813477 31741566

